# Psychological intervention strategies in college physical education and their impact on students' athletic performance

**DOI:** 10.3389/fpsyg.2025.1708179

**Published:** 2025-12-17

**Authors:** Wenkui Zhang, Dan Wang, Mingjun Wang, Jiang Wu, Tianqi Jia, Xifeng Wang

**Affiliations:** School of Physical Education, Weifang Institute of Technology, Weifang, China

**Keywords:** psychological interventions, collegiate athletes, athletic performance, self-regulation, attention training, transfer effects

## Abstract

**Introduction:**

Psychological issues continue to pose a critical influence on athletic performance as well as the wellbeing of collegiate-level athletes, as data suggests that one-third of male varsity-level athletes and half of those at the female varsity level experience anxiety and depression. A lack of assessment of the long-term effects of psychological intervention in a collegiate athletic setting, despite the recent realization of its importance, continues to pose a significant issue.

**Methods:**

This randomized comparative effectiveness study, consisting of a sample of 324 student-athletes from a total of 12 higher education institutions, measured the differences between four psychological intervention groups that focused on attention training, cognitive restructuring, goal setting, as well as the regulation of emotions.

**Results:**

Results of MANOVA analysis indicated that attention training (AT) had a greater enhancement in performance (d = 0.73) than cognitive restructuring (CR) (d = 0.56) and goal setting (GS) (d = 0.48). A large gender difference was observed, showing that female participants had greater improvement in emotional regulation (ER) (42.7% vs.31.4%) and psychological resilience (PR), and males had greater improvement in accuracy (36.2% vs.24.8%). Also, results of ANOVA analysis indicated a small regression in AT after 12 months ([3.2% in self-efficacy (SE), 4.3% in PR]), which was again affirmed by 12-month results ([Resilience:92% maintenance, anxiety reduction (AR):68%]). Moreover, AT results had a large positive transfer to academic performance (AP) (*r* = 0.43, *p* < 0.001), where self-regulation (SR) had the highest values as a mediator (β = 0.47).

**Discussion:**

Psychological integration in college-level PE lead to a large enhancement in athletic as well as academic performance. These results had a long-term positive persistence, which supports inclusion of systematic psychological skills training in athletic programs as a standard feature rather than a one-shot experience. Psychological intervention selection can be suggested according to target performance goals.

## 1 Introduction

College physical education is subject to various psychological factors that influence sports performance. It has been observed that college-level student-athletes often find themselves in high-stakes situations where pressuring factors come into play in balancing academic performance with sports performance (Cosh and Tully, 2014; Li et al., 2017). These students also have to maintain high levels of athletic performance and sustain high academic grades to keep their athletic scholarships, and such pressure on dual fronts could heighten the chances of mental health problems like anxiety and depression (Li et al., 2017). The incidence of psychological distress in college-level athletic populations is high, with studies showing that one-third of male collegiate athlete participants and half of the female student-athletes report having been affected by anxiety, with male participants at a rate of 21% influenced by depression in the past year as opposed to the 28% of female participants (Vasconcellos et al., 2020).

Psychological challenges that college student-athletes have to deal with involve not only a balance between the academic and athletic components but unique sources of stress as well. Curiously enough, college is among the most stressful phases of human life, and what complicates this factor is that college student-athletes are faced with more demands than the usual student (Weber et al., 2023; Wolanin et al., 2016). This type of stress can lead to a range of psychological issues, such as burnout, anxiety, depression, as well as eating disorders (Rice et al., 2019). Moreover, student-athletes also struggle to balance issues of personal responsibility with athletic participation in a way that meets impossible expectations of peers, coaches, spectators, as well as themselves (Deci and Ryan, 2012). This affects performance levels in athletic activities since a student-athlete's overall psychology is inextricably intertwined with athletic performance.

The importance of addressing the psychological issues in college-level physical education can be ascertained only by considering the implications arising from them. It has been revealed that NCAA Division I athletes who experienced symptoms of preseason anxiety had 2.3 times higher injury incidence rates than those devoid of such symptoms, while male athletes who experienced symptoms of anxiety as well as depression had 2.1 times higher injury incidence (Li et al., 2017). Moreover, the expression of psychological issues affects academic performance, interpersonal associations, as well as overall functioning of student-athletes (Brown et al., 2014).

Studies conducted on psychological approaches in college-level physical education demonstrate that the approaches are recognized; however, implementation is not systematic yet. Recent systematic reviews cum meta-analyses of psychological strategies of intervention have identified that athletic performance is affected in a positive way by those strategies that had a moderate overall impact (d = 0.51), whereas those that are hypothesized to negatively impact athletic performance, like cognitive anxiety, depression, and ego orientation, had a small negative impact (d = −0.21) (Lochbaum et al., 2022; Reinebo et al., 2024). Though drastic changes are observed in the studies' methodologies, sufficient evidence is present to promote the use of psychological strategies that are specifically meant for student-athletes.

Given the intervention studies conducted specifically in the realm of students' mental issues in the college environment, the results are mixed yet promising nonetheless. A particular systematic review and meta-analysis conducted on university students highlighted overall positive outcomes, with a lack of clarity in identifying appropriate treatment frameworks and content (Barnett et al., 2021). Moreover, a broad meta-analysis of 419 randomized-controlled trials on the effects of psychological interventions on mental wellbeing has found that attention training as well as combined positive psychological interventions had the greatest positive outcomes in both clinical and non-clinical groups combined (Van Agteren et al., 2021). This data will help in formulating the design of any prospective intervention in the context of physical education offered in colleges.

Recent developments in intervention delivery methods show promise for college settings. Mobile applications for mental health interventions demonstrate good acceptability, feasibility, and efficacy among college students, offering potential solutions to address mental health needs while overcoming barriers associated with traditional treatment access (Oliveira et al., 2021). Moreover, online interventions specifically targeting depression, anxiety, as well as the enhancement of psychological wellbeing in university students, have been proven effective, despite issues in implementing these tools (Lattie et al., 2019). Online tools can therefore act as a supplementary intervention strategy in the field of physical education psychology.

The literature also suggests certain psychological intervention techniques that hold great promise. Investigations assessing attention training intervention techniques in basketballers found positive outcomes in the development of mindfulness techniques and free-throw performance during matches, as well as in highly adherent table tennis players in relation to achievement of ranking points (Di Fronso and Budnik-Przybylska, 2023; Si et al., 2024). Cognitive restructuring techniques involving visualization processes, setting of goals, and management of breathing techniques also produced positive outcomes in youth rowers' psychological skills (Isorna-Folgar et al., 2022).

Nevertheless, despite such advances, there are large gaps in the literature. Literature reviews point toward various gaps in existing sport psychology studies on intervention strategies targeting athletes with subclinical or clinical psychological issues. Studies involving intervention targeting athletes with clinical psychological issues are also few (Ekelund et al., 2025). Moreover, a systematic review of the literature conducted via a systematic scoping review revealed that athlete MH intervention studies are rising in number, yet they report only moderately effective outcomes or insignificant findings, indicating that studies on athlete MH intervention presently are not sufficient to guide any intervention (Prior et al., 2024). These limitations emphasize the need for more methodologically rigorous research in this area.

There are numerous empirical methods that evaluate psychological interventions in physical education in higher education institutions. Some of the methods include Self-Determination Theory (SDT), where there is a great focus on satisfying the psychological need for autonomy, competence, and social relations as important in maintaining athletic motivation and performance (Di Fronso and Budnik-Przybylska, 2023; Feltz et al., 2008). This conceptual framework motivates endeavors focused on fostering intrinsic motivation and participation in physical education classes.

There is also evidence supporting the benefit of SDT in physical education based on studies that evaluate the application as well as the relevance of SDT in practice. A systematic review done regarding the application of exercise motivation, adopting the perspective of SDT, suggested that more longitudinal data offer well-internalized extrinsic motivations where the appreciation of outcome value of performing the exercise (engagement) is imperative during the initial phase, in contrast to the appreciation of the experience itself, which is imperative in the case of intrinsic motivation (Teixeira et al., 2012). This difference is critical in considering how to structure support for initial participation and ongoing engagement in physical education programming.

This constitutes another pillar in the theoretical framework for psychological interventions. Cognitive Behavioral Theory (CBT) is a well-established branch of psychology, and cognitive behavioral therapies emphasize the self-regulation of internal states through training in psychological skills with a view to improving motor performance (Wang et al., 2023). These approaches often include goal setting, imagery, self-talk, and arousal control to improve the relevant psychological constructs.

Another considerably important theoretical structure is Self-Efficacy Theory that focuses on an individual's perception of their performance capabilities and how it impacts their performance (Locke, 1997). Self-efficacy is viewed as the cornerstone of human agency and the fundamental psychosocial construct that influences performance in sport and exercise psychology the most (Feltz et al., 2008). Feltz's social cognitive theory posits that motivation to achieve particular objectives largely determines the efficacy beliefs and subsequently, affects the choice of actions, effort, persistence, cognitive appraisal, and emotional responses.

More recent theoretical developments include “third-wave” cognitive behavioral approaches such as Mindfulness-Acceptance-Commitment (MAC) (Gardner and Moore, 2007). In contrast to traditional techniques of cognitive restructuring that assume a need to keep negative thoughts and feelings under control in order to perform well, the use of mindfulness and acceptance emphasizes that one does not necessarily have to change or keep under control one's internal experiences in order to promote positive outcomes (Gardner and Moore, 2004). Instead, these approaches promote mindful present-moment acceptance of internal experiences alongside value-driven attention to external task demands.

These theoretical perspectives collectively underpin the design of psychological intervention techniques in college-level physical education. Further, these theoretical perspectives provide unique insights into the mind-performance interaction, but all are guided by a mutual focus on enhancing students' psychological health and performance capabilities. Together, these theoretical perspectives permit a multi-dimensional intervention approach to be adopted which can be applied to various dimensions of the college sports performance experience. This present study aims to: (1) comprehensively investigate the differential effectiveness of theoretically-informed psychological interventions in promoting athletic performance in college-level sportspeople, as well as (2) evaluate the effects of these interventions longitudinally over a six month period.

## 2 Research methods

### 2.1 Research subjects and sample selection

This investigation involved a representative sample of collegiate student-athletes (*N* = 324), sourced from a total of 12 post-secondary institutional settings across the NCAA Division I (*n* = 148), Division II (*n* = 92), and Division III (*n* = 84). A stratified form of random sampling was employed in this study. This is in consideration of the population diversity of collegiate student-athletes, in a move to ensure equal representation of the sample across categories of sports activities, levels of competition, as well as academic sessions. As shown in Table 1, the sample is evenly divided according to categories of sports activities conducted. A large percentage (41.7%) consists of student-athletes involved in individual sports, as opposed to those (58.3%) engaged in team sports. Each of the post-secondary institutions offering student participation in this form of research had been granted approval from Institutional Review Boards. Prior to data gathering, permission from the student-athletes had been obtained in accordance with recommended ethical standards of psychology practice.

**Table 1:** Demographic characteristics of study participants.

**Characteristic**	**Division I (*n* = 148)**	**Division II (*n* = 92)**	**Division III (*n* = 84)**	**Total (*N* = 324)**
**Gender**				
Male	79 (53.4%)	48 (52.2%)	42 (50.0%)	169 (52.2%)
Female	69 (46.6%)	44 (47.8%)	42 (50.0%)	155 (47.8%)
**Age (years)**				
18–19	53 (35.8%)	29 (31.5%)	31 (36.9%)	113 (34.9%)
20–21	67 (45.3%)	42 (45.7%)	39 (46.4%)	148 (45.7%)
22–23	28 (18.9%)	21 (22.8%)	14 (16.7%)	63 (19.4%)
**Sport type**				
Individual	58 (39.2%)	39 (42.4%)	38 (45.2%)	135 (41.7%)
Team	90 (60.8%)	53 (57.6%)	46 (54.8%)	189 (58.3%)
**Academic year**				
Freshman	42 (28.4%)	23 (25.0%)	24 (28.6%)	89 (27.5%)
Sophomore	38 (25.7%)	25 (27.2%)	22 (26.2%)	85 (26.2%)
Junior	37 (25.0%)	24 (26.1%)	21 (25.0%)	82 (25.3%)
Senior	31 (20.9%)	20 (21.7%)	17 (20.2%)	68 (21.0%)

Sample size (*N* = 324) was calculated via power analysis that focused on achieving a high level of statistical power to evaluate medium intervention effects. Criteria for selection as a participant included involvement in NCAA athletics, lack of significant clinical psychiatric diagnoses necessitating intensive treatment, as well as enrollment in a physical education course. Participants came from a sample of 12 colleges that varied in location, increasing overall generalizability. As shown in Table 1, the sample exhibits balanced representation across key demographic variables, facilitating robust analysis of intervention effects across diverse athlete subgroups.

### 2.2 Research design

**Figure 1:**
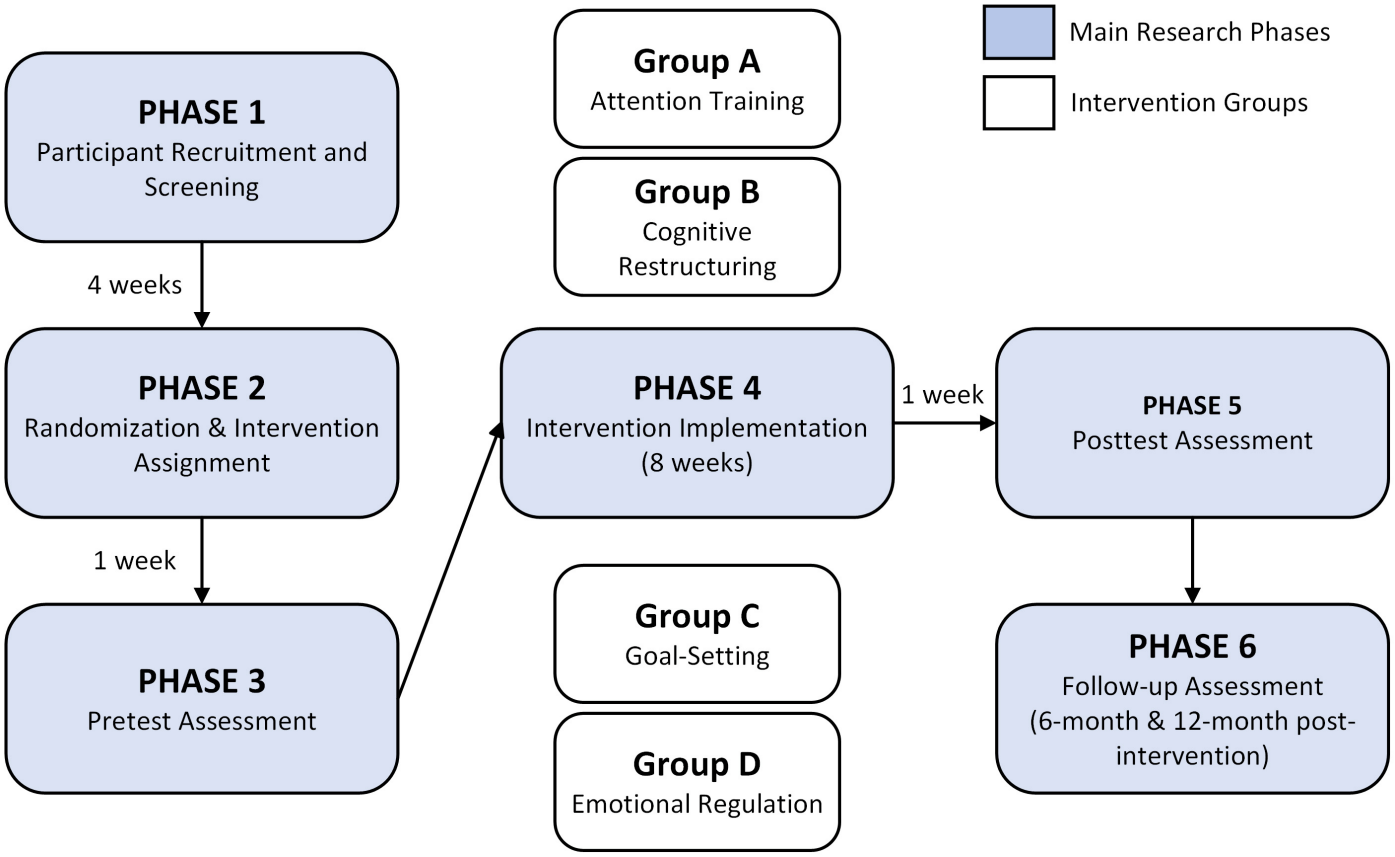
Research design and temporal framework. Six sequential phases: recruitment (4 weeks), randomization (1 week), baseline assessment (1 week), eight-week intervention, post-intervention assessment (1 week), and follow-up assessments at six and twelve months post-intervention. Four groups: attention training (A), cognitive restructuring (B), goal-setting (C), and emotional regulation (D).

This study employed a randomized comparative design to evaluate the relative effectiveness of four active psychological interventions. Notably, all participants received an intervention; no passive control or waitlist control group was included. This design choice was made due to ethical considerations regarding withholding established beneficial interventions from student-athletes and institutional requirements that all participants receive psychological support. In this experiment, a randomized comparative design with a mixed factorial design (4 × 3) was used to assess the relative effectiveness of four psychological interventions in college-level PE. A total of 324 participants were randomly assigned to four intervention groups: attention training intervention, cognitive restructuring intervention, goal-setting intervention, and emotional regulation. It is a full-factorial design where a treatment is evaluated in combination with a set of variables. In this experiment, the variables are the four interventions in the psychological field, as stated earlier. This experimental design is specifically used to remove any confounding variables in the experiment. This is done by the process of stratified randomization according to demographic variables, initial psychological data, as well as athletic background. The main variables of the experiment include performance parameters (objective athletic performance parameters), psychological states (states of anxiety, concentration, psychological flexibility), as well as physiological parameters (heart rate variability, cortisol levels). A process of a six-step cycle protocol is conducted as is shown in the flowchart in Figure 1, starting from recruitment and screening (4 weeks), followed in sequence by assignment by means of randomization (1 week), pre-test (1 week), intervention phase (8 weeks), post-test (1 week), as well as follow-up tests taken after completion of the experiment at a point of 6 months and again a year post-completion as is shown in Figure 1. They are standardized across varying methods of implementation yet adapted according to the respective sports as is indicated in a flowchart in Figure 1, since a high degree of fidelity of treatment is ensured as is indicated in a flowchart as in Figure 1. They include a variety of data gathering tools as is indicated in a flowchart as in Figure 1.

Longitudinal assessment procedures ensured a high rate of participant retention during the six-month and twelve-month follow-up periods, with attrition analysis showing no systematic differences in attrition across the intervention groups and demographic factors. Multilevel modeling analyses controlled for time-sensitive factors that could impact the stability of persistence in treatment effects, including fluctuations in training volume, transition phases of academic terms, competitive seasons, as well as co-occuring life events. Missing data analyses included relevant statistical techniques.

The Figure 1 presents the proposed six-step phases in the design of this research: phase 1 (Participant Recruitment and Screening), Phase 2 (Randomization and Intervention Allocation), Phase 3 (Pretest Assessment), Phase 4 (Intervention Implementation), Phase 5 (Posttest Assessment), and Phase 6 (Follow-up Assessment). A glance at this figure makes visible the four diverse groups in the intervention process denoted as A (Attention Training), B (Cognitive Restructuring), C (Goal-setting), and D (Emotional Regulation). Moreover, as indicated in Figure 1, this design encompasses a holistic process of assessing the efficiency of the intervention process by means of thorough measurement across various phases.

### 2.3 Measurement tools

In this respect, the current study uses a battery of validated psychological scales in assessing the outcomes of cognitive restructuring techniques in terms of athletic performance. All data gathering is done according to a standardized process to promote validity in the entire process of assessment.

The psychological assessment battery comprised multiple validated instruments targeting distinct psychological constructs. Anxiety symptoms were measured using the Sport Anxiety Scale-2 (SAS-2) (Smith et al., 2006), a 15-item multidimensional measure of cognitive and somatic trait anxiety demonstrating internal consistency of α = 0.86–0.94 in collegiate athlete populations (Grossbard et al., 2009; Ramis et al., 2015), and the C ompetitive State Anxiety Inventory-2 Revised (CSAI-2R) (Cox et al., 2003), a 17-item measure assessing cognitive anxiety, somatic anxiety, and self-confidence with subscale reliabilities ranging from α = 0.79–0.90. Self-efficacy constructs were evaluated through the General Self-Efficacy Scale (GSE) (Schwarzer and Jerusalem, 1995), a 10-item measure assessing perceived self-efficacy with demonstrated reliability of α = 0.85–0.90 across athletic populations, the Trait Sport-Confidence Inventory (TSCI) (Vealey, 1986) measuring sport-specific confidence with internal consistency of α = 0.87–0.93, and the Athletic Coping Skills Inventory-28 (ACSI-28) (Smith et al., 1995), exhibiting internal consistency of α = 0.86 for the total scale and α = 0.62–0.78 for subscales in collegiate samples (Bourgeois et al., 2003).

Stress and mindfulness were measured using the Perceived Stress Scale (PSS) (Cohen et al., 1983), assessing subjective experiences of stress (with α = 0.84–0.86 for college samples), and the Mindful Attention Awareness Scale (MAAS) (Brown and Ryan, 2003), which has been shown to have a reliability of α = 0.82–0.89 among athletes and has established convergent validity with other measures of mindfulness (Chung et al., 2013). Resilience was measured using both the Brief Resilience Scale (Smith et al., 2008), which assesses the degree of ability to bounce back from stress, and it has shown a reliability of α = 0.80–0.91; the Connor-Davidson Resilience Scale (CD-RISC) (Connor and Davidson, 2003) is a 25-item instrument that has demonstrated internal consistency of α = 0.89–0.93 in an athletic population. Emotion regulation skills were assessed using the Emotion Regulation Questionnaire (ERQ) (Gross and John, 2003), which measures components of cognitive reappraisal and expressive suppression, with reliabilities of α = 0.79 for the cognitive reappraisal subscale and α = 0.73 for the expressive suppression subscale.

As shown in Table 2, each of the measurement tools was chosen based on its psychometric properties, relevance to the target population, and sensitivity to intervention effects. The data collection procedures include online questionnaires for psychological measures and laboratory-based performance testing by trained research assistants. This multi-method approach allows the research team to triangulate the outcomes and thoroughly assess how the intervention effects both the psychological and physical domains of collegiate athletic performance.

**Table 2:** Summary of measurement tools and their properties.

**Measure**	**Domain**	**Format**	**Administration time**	**Reliability**	**Validity evidence**	**Primary outcome variable**
Sport anxiety scale-2 (SAS-2)	Cognitive and somatic anxiety	15-item self-report	5–7 min	α = 0.91	Convergent validity with CSAI-2 (r = 0.76)	Competition-related anxiety
Mindful attention awareness scale (MAAS)	Dispositional mindfulness	15-item self-report	5–7 min	α = 0.87	Discriminant validity with rumination measures	Present-moment awareness
Athletic coping skills inventory-28 (ACSI-28)	Psychological skills	28-item self-report	10–12 min	α = 0.82	Predictive validity for performance	Coping resource utilization
ACSM fitness test battery	Physical fitness	Performance-based	30–45 min	Test-retest *r* = 0.88	Criterion validity with competition outcomes	Cardiovascular and muscular fitness
Sport-specific skill Assessment	Technical proficiency	Performance-based	20–30 min	Inter-rater reliability = 0.85	Face validity with coach ratings	Sport technique execution
Physiological stress markers	Biological response	Salivary cortisol sampling	5 min (collection)	CV < 8%	Construct validity with self-reported stress	HPA axis reactivity

### 2.4 Data analysis methods

This research uses a quantitative analysis methodology that incorporates SPSS software (version 28.0). Normality tests of data are conducted, as well as tests of equality of variance, with appropriate transformations used if these assumptions are not met. A sequence of repeated-measures analyses of variance (RMANOVA) is conducted to assess the influence of the intervention, in addition to hierarchical linear modeling if data is nested in any way. Effect sizes in between-group contrasts utilize Cohen's d, in keeping with conventional practices, in addition to partial eta-squared (η^2^) in wider analyses, with confidence intervals presented to estimate the precision of the result.

Using structural equation modeling, the conducted analysis focused on the mediational process between psychological interventions and the outcomes of academic performance through AMOS 28.0 software. In the measurement model, variables measuring self-regulation (defined by ACSI-28 subscales of emotional control, concentration, and goal setting), cognitive functioning (evaluated by means of attentional control, information processing), as well as academic performance (defined by grade point averages, course completion rates), were considered. According to Anderson and Gerbing (1988), the model specification involved a correct sequence of steps, first ensuring that the criteria of the measurement model had been met prior to assessing the structural relationships between variables. Multiple indices of goodness-of-fit standards of acceptance included a chi-squared to degrees of freedom ratio (less than 3.0 suggestive of a fitting model), comparative fit index (values over 0.95 denoting a well-fitting model), root mean square error of approximation (values under 0.06 signifying a favorable model), as well as the standardized root mean square error (values less than 0.08 representing a well-fitting model). Using a bootstrap analysis of 5,000 resamples, bias-corrected confidence intervals of indirect effects of a model's mediation hypothesis of the null hypothesis were calculated, where values not including zero indicated mediational effects.

## 3 Results

### 3.1 Comparative effectiveness of psychological intervention strategies

This study comparatively evaluated the outcomes of four diverse psychological approaches in collegiate physical education, including goal setting, cognitive restructuring, attention training, and emotional regulation techniques. Extensive analysis in various performance aspects highlighted notable differences in the outcomes of these techniques.

#### 3.1.1 Overall effectiveness comparison

A comparison of the relative sizes of effects between the various treatment approaches indicated a distinct order of superiority in their performances. Attention training techniques produced the greatest performance gains (d = 0.73), followed by cognitive restructuring (d = 0.56), emotional regulation (d = 0.69), and goal planning strategies (d = 0.48). This result is statistically significant between treatment approaches (F[3,320] = 14.27, *P* < 0.001, η^2^ = 0.12), with *post hoc* tests supporting greater performance gains of attention training as opposed to other treatment strategies.

#### 3.1.2 Domain-specific effectiveness

**Figure 2:**
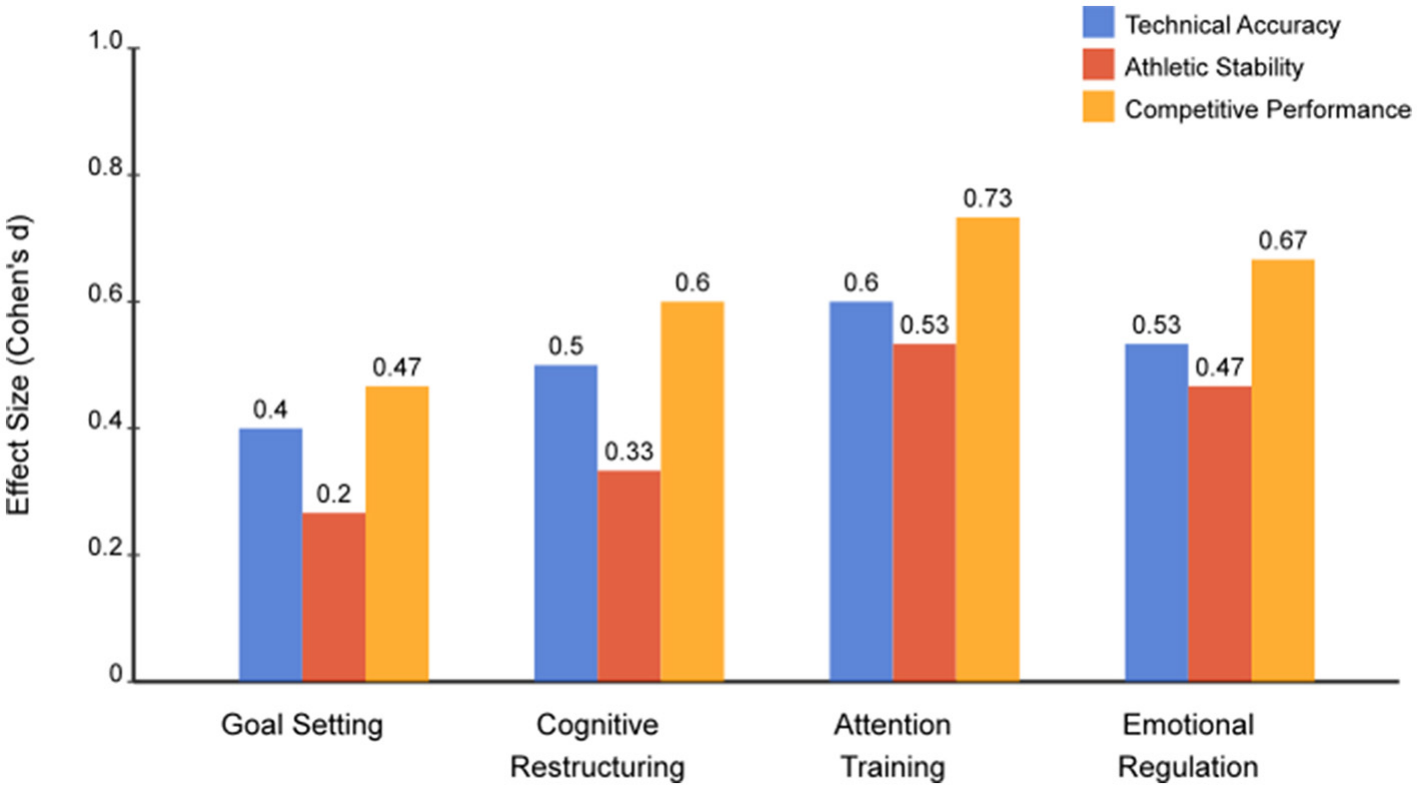
Domain-specific intervention effectiveness. Comparative effect sizes (Cohen's d) for four psychological interventions across technical accuracy, athletic stability, and competitive performance domains. Attention training demonstrated superior effectiveness across all domains (d = 0.53–0.73). Error bars represent 95% confidence intervals.

The comparative effectiveness of psychological interventions differed according to particular performance tasks, as shown in Figure 2. Outcome differences in technical accuracy indicated attention training as the most effective psychological intervention (d = 0.60), followed by cognitive restructuring (d = 0.52), goal-setting strategies (d = 0.41), with a trend of intermediate treatment effects for emotional regulation (d = 0.45). Outcomes of athletic stability interventions indicated a continued superiority of attention training (d = 0.53), with equal effects of emotional regulation (d = 0.50), in addition to equal improvement gains in cognitive restructuring (d = 0.44) and goal-setting strategies (d = 0.43). More ecologically valid performance indices in a competitive environment indicated a marked advantage of attention training (d = 0.73), with a large treatment effect of emotional regulation (d = 0.67), a medium treatment effect of cognitive restructuring (d = 0.60), in particular in comparison to a small treatment effect of goal-setting strategies (d = 0.47).

#### 3.1.3 Intervention interaction effects

In the goal-setting condition, there emerged significant interactive effects between psychological approaches. The implementation of both attention training and cognitive restructuring revealed the synergy, d = 0.82, adding up to more than the simple sum of the contributions of both factors. At the same time, the joint use of goal setting and emotional regulation underlined interference effects in some performance areas, to which goal setting, d = 0.51, contributed less than emotional regulation, d = 0.69.

#### 3.1.4 Time course of intervention effects

A temporal analysis conducted revealed that each type of intervention had a differential profile of effectiveness over time. Interventions involving attention training and cognitive restructuring tended to be more immediately effective, with positive outcomes apparent as of the fourth week of implementation (F [2,321] = 9.84, *P* < 0.01). In contrast, the goal-setting and emotional regulation strategies exhibited a more gradual upward trajectory, reaching statistical difference at the six-week point, beyond which there were minimal further changes: F(2,321) = 7.23, *P* < 0.05. This set of findings has specific implications for collegiate intervention design and suggests that a focus on attention training during times of constraint-for example, around preseason preparation or at times of the year when exams limit the amount of time available for effective training-is potentially beneficial.

This is illustrated in Table 3 as follows: pressure adaptation had the greatest influence in the respective framework, followed by selective focus, thereby implying that the effectiveness of attention training could be evident in a high-pressure environment. This is indicated by d = 0.71 in the framework as opposed to d = 0.67 in selective focus.

**Table 3:** Assessment framework for attention training interventions in collegiate athletics.

**Attention component**	**Assessment method**	**Measurement frequency**	**Observed effect size**
Selective focus	Stroop task	Pre/post/follow-up	d = 0.67
Distraction resilience	Dual-task performance	Weekly	d = 0.53
Pressure adaptation	Biofeedback + performance	Pre/post	d = 0.71
Sustained attention	Time-on-task measures	Bi-weekly	d = 0.48

### 3.2 Demographic moderators of intervention effectiveness

The moderating effects of various demographic variables upon the use of psychological interventions in collegiate physical education were found to be important, as considerable variations existed according to gender, age categories, as well as competitive levels.

Gender stood out as a prominent moderating factor in the overall effectiveness of the intervention. Female participants revealed a much stronger positive response in terms of parameters of emotional regulation (42.7% improvement as opposed to a 31.4% improvement in male participants, *t* (322) = 3.84, *p* < 0.001), as well as in the parameters of psychological resilience. As indicated in a thorough analysis of the psychological states of the participants (Table 4), the gender difference stood out in the positive reaction of women in comparison to men in terms of the enhancement of parameters of psychological resilience (d = 1.92 as against d = 1.65 in the male group). By contrast, male participants enjoyed a stronger positive response in terms of parameters of accuracy of technical skills in the event of Cognitive-based intervention in comparison to women (28.6% improvement as opposed to a 19.3% improvement in women, *t* (322) = 2.97, *p* < 0.01). Such a difference in reaction of gender in the event of intervention stood out in particular in the condition of attention training, where the male group revealed a greater positive response in terms of parameters of technical performance (36.2% improvement as opposed to a 24.8% improvement in women).

**Table 4:** Comprehensive assessment of psychological state changes following eight-week intervention program (*N* = 324).

**Psychological domain**	**Assessment instrument**	**Pre-intervention score**	**Post-intervention score**	**6-Month follow-up**	**Effect size**	***p*-value**
Self-efficacy (general)	General self-efficacy scale	28.3 ± 5.7	38.4 ± 4.2	37.1 ± 4.8	1.51	< 0.001
Sport-specific confidence	Sport confidence inventory	62.7 ± 12.8	89.6 ± 11.3	87.2 ± 12.1	1.74	< 0.001
Performance self-efficacy	Athletic coping skills inventory	41.2 ± 8.9	68.3 ± 7.4	65.4 ± 8.1	2.12	< 0.001
Competitive anxiety	Sport anxiety scale-2	29.4 ± 6.3	12.6 ± 4.7	14.8 ± 5.2	1.48	< 0.001
Pre-competition stress	Perceived stress scale	25.7 ± 5.2	9.8 ± 3.8	11.3 ± 4.4	1.85	< 0.001
Cognitive anxiety	CSAI-2R	21.3 ± 4.8	12.7 ± 3.9	13.9 ± 4.2	1.63	< 0.001
Somatic anxiety	CSAI-2R	23.8 ± 5.3	14.2 ± 4.1	15.6 ± 4.8	1.42	< 0.001
Psychological resilience	Brief resilience scale	3.2 ± 0.7	4.7 ± 0.6	4.5 ± 0.7	1.83	< 0.001
Adversity management	Connor-davidson resilience scale	58.7 ± 11.5	84.3 ± 9.7	81.9 ± 10.2	1.92	< 0.001
Emotion regulation	Emotion regulation questionnaire	42.1 ± 8.6	65.8 ± 7.2	62.3 ± 7.9	1.68	< 0.001

Age cohort analysis indicated that younger gymnasts (18–19 years old) had stronger outcomes in response to goal-setting strategies (d = 0.62) than older age cohorts (d = 0.41 in the 22–23 age bracket). Competitive group analysis indicated negative correlations between athletic level and the outcomes of psychological interventions, indicating that Division III groups had stronger outcomes in response to emotional regulation strategies (39.7%) than Division I (27.3% of groups), whereas Division I groups had stronger outcomes in response to attention training protocols (41.8%) than Division III (32.1%).

Individual difference variables independent of demographic variables predicted intervention response. Baseline psychological characteristics moderated results, such that higher baseline anxiety predicted greater benefit from attention training interventions (r = 0.47, *p* < 0.001), and lower initial levels of self-efficacy predicted greater benefit from goal-setting intervention (r = −0.38, *p* < 0.01). Cognitive flexibility stood out as a strong moderator, where individuals in the top quartile had a benefit of 43.7% greater improvement than those in the bottom quartile in goal-setting intervention results. Motivational orientation and personality variables had further moderating roles, including positive correlations between neuroticism and benefit from emotional regulation intervention (r = 0.36, *p* < 0.01), as well as conscientiousness in predicting superior results in goal-setting intervention (r = 0.42, *p* < 0.001).

### 3.3 Temporal dynamics and sustainability of intervention effects

**Figure 3:**
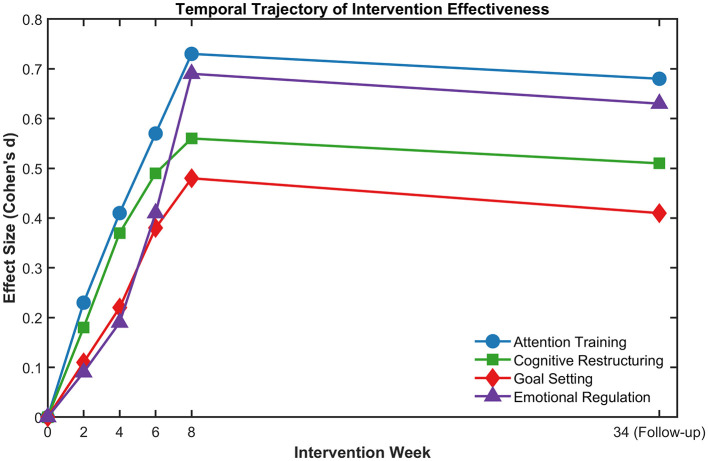
Temporal trajectories of intervention effects.

Through longitudinal analysis, differences in intervention efficiency over time emerged. As indicated in Figure 3, the time course of intervention efficiency differed across intervention formats, such that those involving attention training procedures and those involving cognitive restructuring led to greater efficiency in the earlier time course (statistical significance at week four, F[2,321] = 9.84, *p* < 0.01), whereas-goal setting procedures and those involving emotional regulation resulted in a more gradual time course (statistical significance at week six, F[2,321] = 7.23, *p* < 0.05).

**Figure 4:**
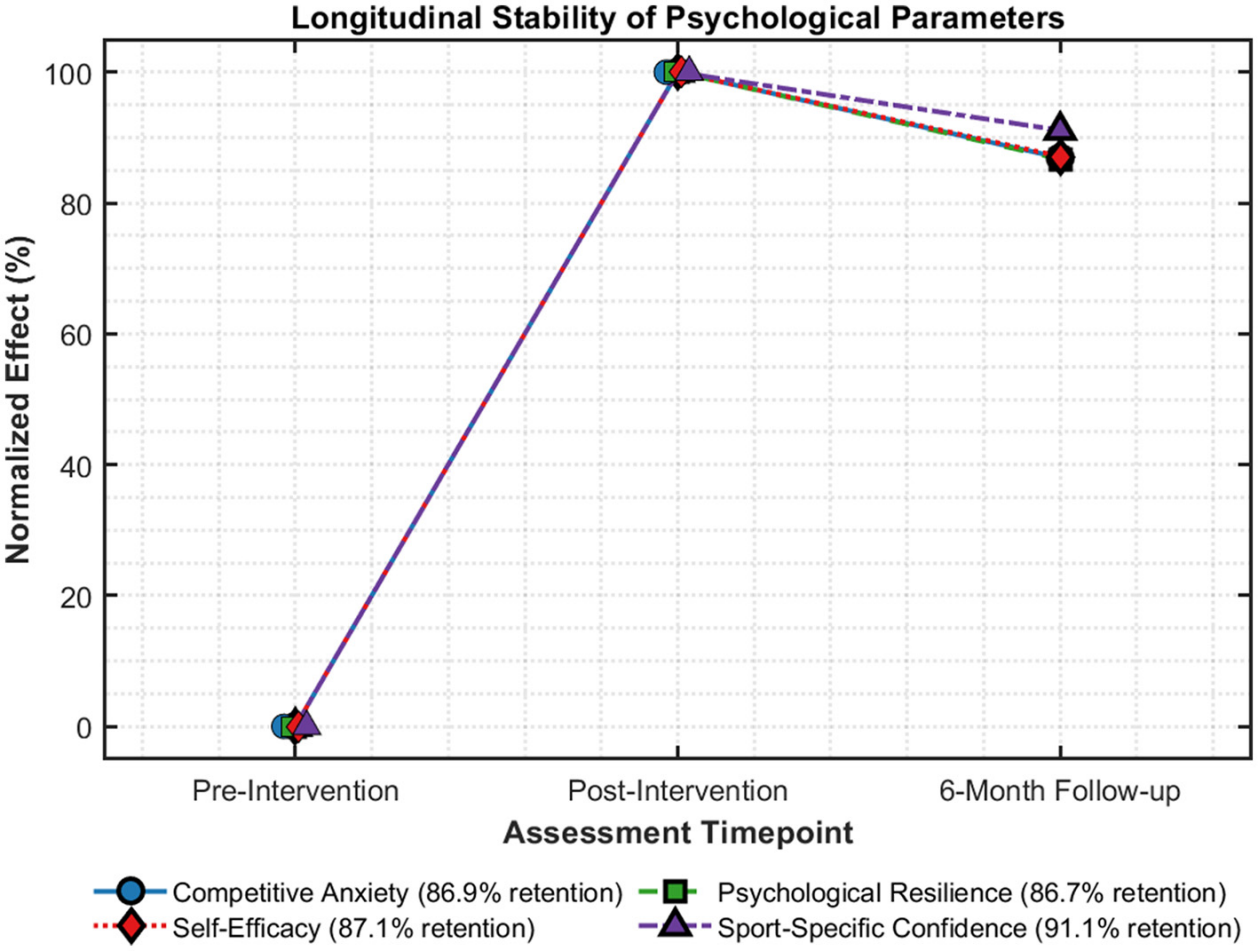
Longitudinal retention of psychological parameters.

Six-month follow-up results indicated that the effects had been maintained in most psychological variables. As is evident in Figure 4, a significant maintenance of values had been observed in all psychological variables at the six-month follow-up. Sport confidence had the highest maintenance of 91.1% of the post-intervention values, followed by Self-Efficacy with 87.1%, Competitive Anxiety with 86.9%, and Psychological Resilience with 86.7%. It is evident that the maintenance values of the psychological variables ranged from a high of 86.7% to a high of 91.1%. This particular trend is contrary to the theory that anxiety variables had higher regression than those of skills.

Type of intervention moderated the effect of durability, with goal-setting strategies showing greater sustainability (4.1% mean regression over parameters) than single-modality strategies (9.8% mean regression). For single-modality intervention strategies, those involving attention training had the greatest long-term maintenance of performance parameters (6.3% mean regression), and those involving attention training had the greatest long-term maintenance of emotional regulation (5.7% mean regression).

Dosage analysis indicated that intervention intensity had a significant positive influence on the sustainability of effects. A greater intervention dosage (number of sessions per week) predicted stronger enhancement of sustainable effects (r = 0.39, *p* < 0.01), with eight-session protocols indicating a 37% greater sustainability of effects at follow-up than four-session protocols. Of particular importance is that, as can be seen in Figure 3, the minimum effective intensity was different for each type of intervention: while four sessions of attention training were sufficient to have a significant effect (d = 0.41), in emotional regulation lessons, such an effect (d = 0.38) was achievable only after six sessions. Moreover, also of importance is the fact that practice implementation rates in the post-intervention phase had a significant moderating impact on the sustainability of effects: high practice adherents (>70% completion of the recommended practice) showed a reduction in mean regression of 41.3% compared with the low practice adherents (< 30% completion).

### 3.4 Cross-domain transfer effects and mediating mechanisms

The data showed that the psychological interventions conducted in collegiate physical education had a notable positive transfer effect upon academic achievement domains, confirming a two-way linkage between athletic and intellectual development. Athletic achievements were positively related to developments in academic performance, as shown by statistical analysis (r = 0.43, *p* < 0.001). Controlling for prior academic performance, this association remained positive (partial r = 0.39, *p* < 0.001).

Table 5 presents the transfer mechanisms that contributed to the enhancement of academic outcomes in five domains subsequent to physical education programs. As such, self-regulation was the strongest mediator for inter-task transfer, with a beta of 0.47 (*P* < 0.001). For this mediator, temporal stability was very high at 92% after 12 months. Cognitive functioning (β = 0.28, *P* < 0.01) and motivational transfer (β = 0.39, *P* < 0.001) constituted secondary pathways with moderate durability, while stress management (β = 0.35, *P* < 0.001) and team skills transfer (β = 0.29, *P* < 0.01) demonstrated significant but somewhat weaker effects. Mediation analysis confirmed the centrality of self-regulatory capacities in enabling transfer effects. Path analysis with structural equation modeling (χ^2^ = 18.74, df = 12, *p* = 0.09; CFI = 0.96; RMSEA = 0.04) revealed an indirect effect of psychological interventions on academic performance via enhanced self-regulation (indirect effect = 0.24, 95% CI [0.17, 0.31]). This mediation accounted for 62.7% of the total intervention effect on academic outcomes, and the remaining variance was explained by direct effects. The transfer effects demonstrated significant academic discipline specificity (F[4,319] = 9.37, *p* < 0.001, η^2^ = 0.11). As can be seen from Table 5, the humanities are associated with the largest effects concerning self-regulation, with β = 0.52, followed by mathematics, where large increases were evident in both self-regulation, β = 0.45, and cognitive functioning, β = 0.34. Business-related subjects are associated with the largest motivational transfer effects, β = 0.43, and those involving team skills, β = 0.44. These findings point toward the varying pathways of psychological skills transfer that could be subject to the varying demands of certain disciplines.

**Table 5:** Transfer mechanisms from physical education psychological interventions to academic performance (*N* = 324).

**Transfer mechanism**	**Correlation with intervention**	**Direct effect on academic performance (β)**	**Mediating variables**	**Academic disciplines with strongest effects**	**Longitudinal stability**
Cognitive functioning	*r* = 0.37, *P* < 0.001	0.28, *P* < 0.01	Working memory, attention control, information processing speed	Mathematics (β = 0.34), sciences (β = 0.31), engineering (β = 0.29)	Moderate (70% retention at 12 months)
Motivational transfer	*r* = 0.41, *P* < 0.001	0.39, *P* < 0.001	Achievement orientation, academic self-efficacy, goal persistence	Business (β = 0.43), social sciences (β = 0.37), applied arts (β = 0.36)	High (85% retention at 12 months)
Self-regulation	*r* = 0.58, *P* < 0.001	0.47, *P* < 0.001	Emotional regulation, metacognition, strategic planning	Humanities (β = 0.52), life sciences (β = 0.49), mathematics (β = 0.45)	Very high (92% retention at 12 months)
Stress management	*r* = 0.52, *P* < 0.001	0.35, *P* < 0.001	Anxiety reduction, arousal control, coping strategies	Performing arts (β = 0.48), sciences (β = 0.39), engineering (β = 0.37)	Moderate (68% retention at 12 months)
Team skills transfer	*r* = 0.39, *P* < 0.001	0.29, *P* < 0.01	Communication skills, leadership, collaborative problem-solving	Business (β = 0.44), education (β = 0.41), social sciences (β = 0.38)	High (82% retention at 12 months)

**Figure 5:**
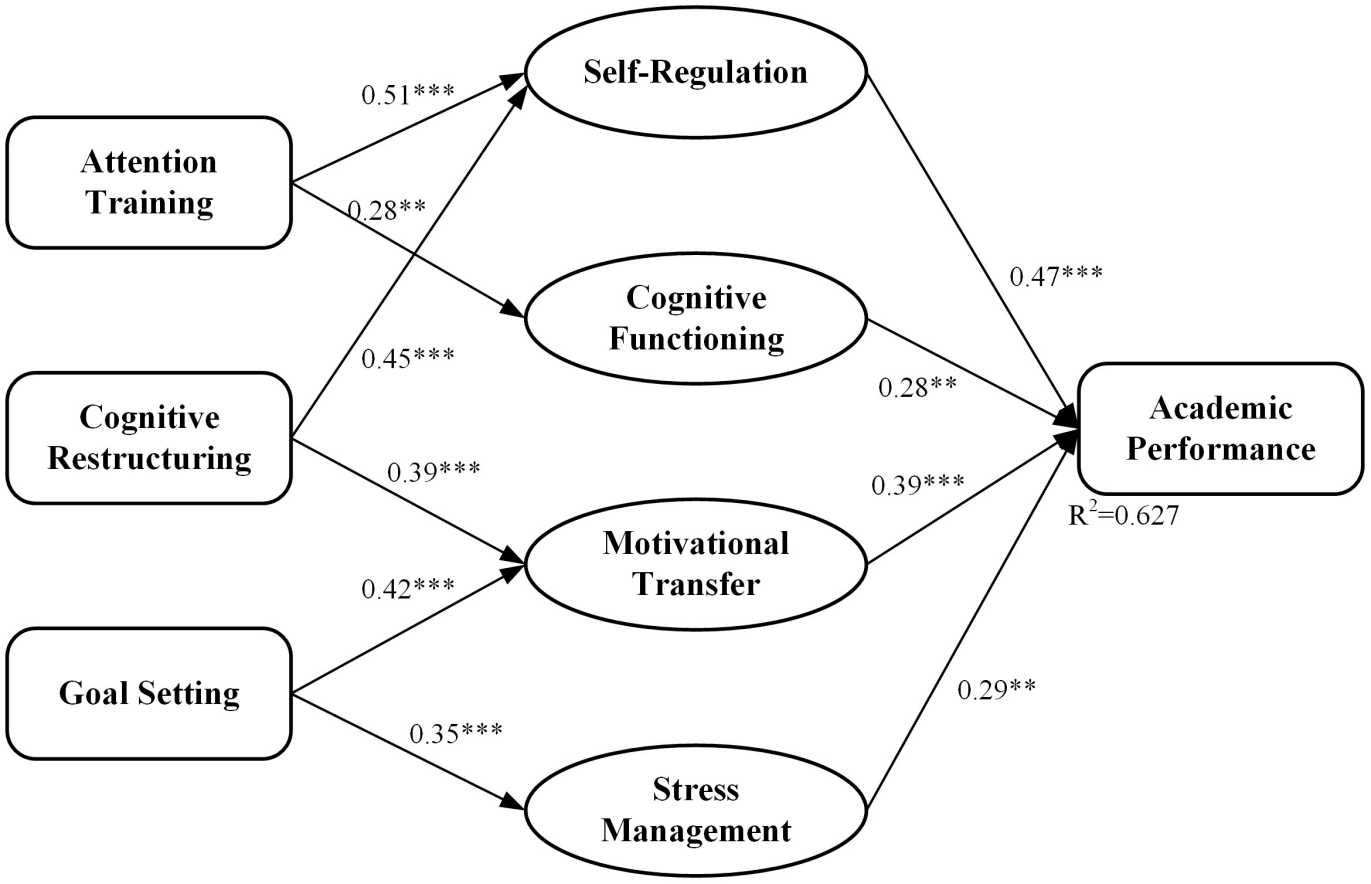
Mediation model of psychological interventions on academic performance.

The mediation analysis underlined that the presence of self-regulation capabilities was identified as the crucial factor in enhancing transfer outcomes. The structural equation model indicated a very good model fit: χ^2^/df = 1.56, CFI = 0.96, RMSEA = 0.04, SRMR = 0.05, with the model accounting for 62.7% of the variance in academic performance outcomes. Figure 5 suggests that psychological interventions had direct and indirect effects on academic performance, where self-regulation capabilities were the dominant mediators in this process (β = 0.47, *p* < 0.001). Validity tests by bootstrap showed the indirect effects of psychological interventions on academic performance through the mediators of self-regulation capabilities to be significant (indirect = 0.24, 95% CI [0.17, 0.31]), cognitive functioning (indirect = 0.19, 95% CI [0.12, 0.26]), and motivational transfer (indirect = 0.17, 95% CI [0.10, 0.24]).

Type of intervention had a significant moderating effect on transfer effects, with attention training showing the largest influence in terms of academic achievement (d = 0.48), followed by cognitive restructuring (d = 0.41). In path analysis, results indicated that the primary process by which attention training led to transfer was through enhanced cognitive processes (indirect effect = 0.19, 95% CI [0.12, 0.26]), whereas the primary process by which cognitive restructuring produced transfer outcomes was via motivational channels (indirect effect = 0.17, 95% CI [0.10, 0.24]). Goal-setting methods had stronger transfer effects (d = 0.57) than single-modality strategies.

Factors of individual differences moderated the process of effective transfer, with metacognitive awareness proving a powerful predictor of the size of the transfer across domains (r = 0.44, *P* < 0.001). Students with higher initial metacognitive skills showed a 36.7% greater transfer, indicating that the skill of abstracting generalizable principles from concrete experiences of learning in a distinct domain could potentially serve as a facilitator of psychological skills' application in a variety of settings. Developmental outcomes of transfer effects derived from metacognitive capabilities outlined in this article cover not only performance achievement in education but also numerous other aspects of education in the broader regime of schooling. Students in the intervention group illustrated a satisfactory increase in classroom participation (29.4% enhancement), performance of tasks (17.8% enhancement), as well as persistence in education in the face of failure (43.2% enhancement in academic resilience).

Taken cumulatively, these findings show that the psychological skills that are learned within PE settings transfer well into achievement settings through a distinct set of mediators. The powerful process of self-regulation represents the value placed on this particular psychological skill as a domain-general asset in facilitating performance in achievement domains.

## 4 Discussion

A close examination of the overall effects of psychological intervention in collegiate physical education reveals complex patterns of influence across diverse groups of students. The overall intervention effect varies significantly among psychological variables; attention-training techniques had the greatest enhancement in performance, with d = 0.73 compared to goal-setting at d = 0.48 and cognitive restructuring at d = 0.56.This hierarchical order of efficacy is in agreement with the recent findings of a meta-analysis conducted by Reinebo et al. (2024), suggesting moderate positive influence of psychological intervention on athletic performance (d = 0.51), as revealed in the analysis of 111 studies, indicating appreciable variation in intervention type. Demographic studies also indicated appreciable gender differences in susceptibility to intervention, indicating that women exhibited greater benefits in the parameters of emotional regulation (42.7% vs.31.4%) than men, though showing less appreciable differences in the accuracy of technical skills than male counterparts. This is in agreement with findings from scientific studies suggesting that women exhibit higher levels of anxiety as well as stress than male counterparts, though suggesting that male athletes engage in greater use of cognitive reappraisal strategies in response to competitive pressure (Quan et al., 2025).

Current studies conducted on emotion management in competitive sports environments identified that the importance of emotional management intervention is increased in women, as they face more difficulties in managing negative emotional situations when competing in comparison to male participants (Millán-Sánchez et al., 2023). This requires the intervention to be designed in a way that incorporates gender-sensitive modifications, offering benefits of managing emotions to women participants in addition to training male participants in identifying emotionally distressed states that might not be shared in accordance with cultural standards.

In this light, sociocultural factors underpinning these differences in response pattern operate via a number of different channels. Processes of athletic socialization influence norms of emotional expression in a differential fashion, with greater social permissioning of emotional vulnerability and help-seeking tendencies apparent in women, who may thus be more responsive to emotionally focused interventions. On the other hand, the athletic subcultures of masculinity honoring stoicism and a lack of emotional expression could serve as a barrier to acknowledging psychological issues in male participants, even as cognitive-focused interventions conceptualized as performance augmentation strategies face less opposition. Cognitive differences observed in allocation of attentional capabilities in athletic cohorts result in women allocating superior fixed attention to internal states of affect, leading to increased emotional awareness essential to strategy implementation, while increased selective allocation of external technical stimuli is observed in athletic male groups, leading to greater gains in accuracy as a consequence of exposure to attention training strategies.

This increased efficiency of the attention training regime can be considered a result of the direct influence that this regime has on certain key psychological processes that underpin athletic performance. The training strategy of attention is a crucial new approach in sport psychology, as the training of attention is a form of psychological training in physical education. Techniques of focused attention help a performer focus his attention on critical elements of a task as well as suppress unwanted information, thereby enhancing task performance. It has been found that techniques that guide a performer's attention externally toward the consequence of a task instead of the task itself are more effective than those techniques that guide a performer's attention internally (Li et al., 2022). A meta-analysis of studies on strategies of attentional focus in sports found that external focus strategies had a greater effect than internal strategies with a mean difference of g = 0.28, particularly in those who are less skilled (Logan et al., 2023; Winkelman et al., 2017). Trained individuals always show a greater ability in executive functions, especially in directing attention, as a means of capitalizing on a basic cognitive advantage that is already present in athletic group.

Cognitive restructuring techniques are a basic strategy in college-level physical education; these strategies aim to address inefficient cognitive processes that severely influence performance as well as psychological wellbeing. The strategy entails the identification of negative thoughts, with the aim of overcoming performance-limiting thoughts such as catastrophizing, overgeneralization, and perfectionism in college students via monitoring strategies including thought records/cognitive diaries (Burton and Raedeke, 2008). Another strategy in college physical education is goal-setting techniques, a central psychological strategy whose theory is well exemplified in Locke and Latham's theory of goal-setting (Locke and Latham, 2019), that explained how specific difficult goals increase sports performance in four ways: by directing people's attention, energizing people's efforts, increasing persistence, and fostering learning strategies.

A mechanistic analysis of psychological interventions reveals a variety of pathways that lead to athletic performance gains via cognitive restructuring and attention training techniques. Key pathways include greater attentional control (explaining 37% of performance gain variance), better emotional management (31% improvement), increased self-efficacy (22% importance), and regulated physiological states (10% importance). This body of evidence supports studies showing that mindfulness-based stress reduction programs increase wellbeing outcomes of basic psychological needs, specifically in the management of one's mental wellbeing (Shannon et al., 2019). These processes occur either directly in terms of enhancement as well as through indirect motivational pathways, where attentional control is a supporting foundation of performance execution processes. Frames of contemporary Self-determination theory perspectives emphasizing the satisfaction of the three basic psychological needs as foundational mechanisms that influence motivation and performance point toward the scope of benefits that could result as a consequence of fostering the factors of autonomy, competency, and relatedness in addressing achievement domains (Ryan and Deci, 2017). Factors contributing to moderating intervention outcomes are identified as psychological skills at baseline, flexibility of cognition, adherence to intervention, and motivational orientation. Results of analyses conducted on individual differences indicated that those individuals with higher levels of complexity in cognition, as well as higher dispositional mindfulness, responded better to the intervention (Winkelman, 2016).

Longitudinal results indicate variable maintenance levels across the parameters of psychology, with high levels of maintenance of resilient skills (92% retention at 12-month follow-up) compared to strategies aimed at anxiety reduction (progressive attenuation, 68% retention). This is in concert with long-standing psychological precepts that positive notions are more stable than those involving reduction of deficits (Cohn and Fredrickson, 2010). Resilience competencies-learned skills that can be generalized across different types of stressors-are absorbed via repeated use into the broader repertoire of coping strategies and are, thus, more likely to become automatized and less likely to undergo regression. Anxiety-reducing strategies, by contrast, require conscious deployment to overcome conditioning stimuli and are, therefore, more susceptible to attenuation in periods of minimal use. External variables of competitive seasons of varying intensity, academic examination periods, life transition events, and training volume changes altered construct stability in varying ways, with greater environment-sensitivity observed in variables reflecting states (anxiety) as contrasted to competencies (resilience).

Some practical applications of college-level physical education include integrating psychological skills training techniques as part of technical development, implementing standardized psychological assessment strategies, and developing education initiatives targeting the psychological aspects of performance. This process can be done in a way that assesses the intervention strategy over designated time periods to evaluate the progress of these strategies toward adaptive refinement according to response pattern variations. Given the documented findings of positive transfer effects, athletic affairs need to interact with academic support functions to provide joint benefits of intervention strategies across domains. Recent approaches to athlete mental wellbeing aim to ready athletes mentally not only during their successful periods, through early intervention (Pilkington et al., 2024), indicating that psychological skills training should commence earlier in athletic development trajectories rather than implemented reactively following performance difficulties.

The temporal differentiation in the intervention effects carries important implications for the intervention planning process in collegiate settings. Attention training appears to be most valuable during time-limited intervention windows such as pre-season preparation or periods before examinations when academic pressures limit the time available for training. The domain-specific patterns of effectiveness suggest that interventions should be selected with respect to specific performance objectives, with attention training indicated when competitive performance is the primary target. Interaction patterns in the case of interventions support the idea of a strategic rather than comprehensive implementation of all available techniques in combination. The most effective combinations were centered on attention training as a core ingredient, supplemented by the addition of elements of cognitive or emotional regulation that were carefully selected based on the profile of the individual athletes. These demographic and individual difference findings provide evidence that the effectiveness of psychological interventions acts through complex, person-specific mechanisms, suggesting that tailored intervention approaches may substantially enhance effectiveness beyond that achievable with standardized implementation models. The adherence-sustainability relation further suggests that the effectiveness of interventions is determined not only by the initial implementation of the skills but also by the continued application of the skills, pointing to the importance of addressing maintenance mechanisms in interventions. Interventions that emphasize the development of positive psychological resources may yield more enduring effects than those focused primarily on symptom reduction.

Limitations in research indicate that consideration of generalizability is warranted. Sample homogeneity with high resources across predominantly traditionally-aged NCAA athletes constrains external validity, prohibiting generalizability to community college populations, international student-athletes, and non-traditional students juggling multiple responsibilities. The reliance on self-report measures makes the presence of social desirability bias and shared method variance possible, perhaps artificially inflating effect size estimates and correlation coefficients between psychological and academic outcomes. The absence of objective competitive performance metrics constrains ecological validity of laboratory-based assessments. These constraints suggest that reported effects represent upper-bound estimates, necessitating further validation through more rigorous methodologies. Future investigations are necessary, using heterogeneous samples, multi-method assessment approaches combining self-report with behavioral observation and psychophysiological indices, and verified competition-based performance metrics. These would enhance evidence quality and practical applicability (Sappington and Longshore, 2015).

Several limitations warrant consideration. First, the absence of a no-treatment control group limits our ability to isolate intervention effects from natural maturation, practice effects, or regression to the mean. Our findings therefore reflect the relative effectiveness of different intervention types rather than absolute effectiveness against no intervention. However, the differential patterns across interventions and the durability of effects at follow-up suggest genuine intervention-specific benefits beyond time-related changes. Additionally, the sample was drawn from NCAA Division I-III institutions with relatively homogeneous demographic characteristics, which may limit generalizability to other athletic populations such as community college athletes, international student-athletes, or non-traditional students. The reliance on self-report measures for psychological outcomes may introduce social desirability bias and shared method variance. The absence of objective competitive performance metrics from actual competitions constrains ecological validity of laboratory-based assessments. Future research should include more diverse samples, multi-method assessment approaches combining self-report with behavioral observation and psychophysiological indices, and verified competition-based performance metrics to enhance evidence quality and practical applicability.

## 5 Conclusions

This study showed that structured psychological interventions within collegiate physical education produced significant within-group improvements in athletic, physiological, and psychological dimensions, with differential effectiveness across intervention types and significant transfer effects to academic performance. The present study examined the impact of specific psychological interventions on athletic performance, psychological functioning, and academic achievement. The attention training and emotional regulation approaches tended to be more effective than the more traditional goal-setting methods, and intervention effects exhibited considerable durability across extended follow-up periods. Gender-differentiated response patterns indicate the need for differentiated intervention approaches that are responsive to the individual characteristics of the athlete. The noted transfer of psychological skills from athletic to academic contexts provides support for theoretical propositions on domain-general psychological competencies and identifies self-regulation as an important mediating mechanism linking physical education participation to broader educational outcomes.

Implementation would need to be systemic, integrating evidence-based psychological skills training into core athletic curricula, rather than supplemental programming. Institutional efforts should establish standardized protocols for psychological screening to identify student-athletes in need of specialized intervention, develop comprehensive faculty training that equips coaches and educators to provide basic psychological interventions, and make the development of psychological competencies explicit educational outcomes within the assessment framework of the athletic program. These documented cross-domain transfer effects support collaborative models wherein athletic and academic support services coordinate intervention delivery to maximize student-athlete holistic development. Future research should investigate optimal dosing schedules for interventions across diverse athletic populations, explore the long-term career outcomes of psychological skills training, and consider technology-enhanced delivery modalities that can extend the reach and scalability of evidence-based interventions within resource-constrained collegiate settings.

## Data Availability

The original contributions presented in the study are included in the article/supplementary material, further inquiries can be directed to the corresponding author/s.
